# High-Throughput Isolation of Giant Viruses in Liquid Medium Using Automated Flow Cytometry and Fluorescence Staining

**DOI:** 10.3389/fmicb.2016.00026

**Published:** 2016-01-29

**Authors:** Jacques Y. B. Khalil, Stephane Robert, Dorine G. Reteno, Julien Andreani, Didier Raoult, Bernard La Scola

**Affiliations:** ^1^Centre National de la Recherche Scientifique 7278, Institut National de la Santé et de la Recherche Médicale U1095, Unité de Recherche sur les Maladies Infectieuses et Tropicales Emergentes, UM 63, IRD 198, Facultés de Médecine et de Pharmacie, Aix Marseille UniversitéMarseille, France; ^2^Vascular Research Center of Marseille, Institut National de la Santé et de la Recherche Médicale, Faculté de Pharmacie, UMR-S1076, Aix-Marseille UniversitéMarseille, France; ^3^Institut Hospitalo-Universitaire, Méditerranée Infection, Pôle des Maladies Infectieuses et Tropicales Clinique et Biologique, Fédération de Bactériologie-Hygiène-Virologie, Centre Hospitalo-Universitaire Timone, Assistance Publique – Hôpitaux de Marseille and Aix Marseille UniversitéMarseille, France

**Keywords:** giant viruses, protozoa, flow cytometry, high-throughput, automated system, gating strategy, fluorescence staining

## Abstract

The isolation of giant viruses using amoeba co-culture is tedious and fastidious. Recently, the procedure was successfully associated with a method that detects amoebal lysis on agar plates. However, the procedure remains time-consuming and is limited to protozoa growing on agar. We present here advances for the isolation of giant viruses. A high-throughput automated method based on flow cytometry and fluorescent staining was used to detect the presence of giant viruses in liquid medium. Development was carried out with the *Acanthamoeba polyphaga* strain widely used in past and current co-culture experiments. The proof of concept was validated with virus suspensions: artificially contaminated samples but also environmental samples from which viruses were previously isolated. After validating the technique, and fortuitously isolating a new Mimivirus, we automated the technique on 96-well plates and tested it on clinical and environmental samples using other protozoa. This allowed us to detect more than 10 strains of previously known species of giant viruses and seven new strains of a new virus lineage. This automated high-throughput method demonstrated significant time saving, and higher sensitivity than older techniques. It thus creates the means to isolate giant viruses at high speed.

## Introduction

The discovery of Mimivirus opened a new era in virology (La Scola et al., 2003). The description and genome analysis of this giant virus revealed the presence of numerous genes of eukaryotic and bacterial origins that suggests new metabolic activities for viruses (Raoult et al., 2004). Recently, it was proposed that the virus be classified under a new order Megavirales (Colson et al., 2012). Other members of this order are pathogens of animals and several unicellular eukaryotes. Mimivirus deserved some special attention, not only because it was larger than the largest virus known before but also because it was the first virus having dimensions (particle size and genome complexity) that significantly overlap with those typical of parasitic cellular microorganisms. The recent discovery of Pandoraviruses and Pithovirus revealed the extremely large particle size and genetic contents that viruses could possibly possess (Philippe et al., 2013; Legendre et al., 2014). The study of giant viruses is of interest to multiple fields, including environmental studies aimed at understanding the ubiquity of these viruses, their diversity, and their roles in evolution and ecosystem changes (Suttle, 2007; Monier et al., 2008a,b; Claverie et al., 2009). In addition, giant viruses are also of interest in clinical research to study their potential pathogenicity and their impact on human health (La Scola et al., 2005; Khan et al., 2006; Raoult et al., 2006, 2007; Ghigo et al., 2008; Vincent et al., 2009; Mueller et al., 2013; Saadi et al., 2013). There is a need to discover new strategies capable of isolating giant viruses and intensifying studies on these viruses. Finally, improved knowledge on these giant viruses may also shed some light on the evolution of eucaryotes since Marseilleviridae exhibit histone-like proteins (Thomas et al., 2011). Considering that giant viruses are pathogens for protozoa (Abrahão et al., 2014) and cause lysis especially of adherent protists, the amoeba co-culture method became the tool allowing the isolation of these giant viruses (La Scola et al., 2010; Pagnier et al., 2013; Philippe et al., 2013; Legendre et al., 2014). However, this original technique remains fastidious and time-consuming. A recently proposed system for virus isolation on agar plates called the lysis plaque assay allowed hundreds of samples to be tested in a limited time (Boughalmi et al., 2012). This technique, however, has a major drawback that it is limited to the use of adherent amoeba and protozoa with limited mobility, particularly *Acanthamoeba* sp. Furthermore, among the giant viruses, CroV was isolated with a highly motile marine protozoa *Cafeteria roenbergensis* (Fischer et al., 2010), demonstrating the need for a high-throughput isolation system in liquid media. To use a wider range of protozoa, and to screen the largest number of samples, we modified our isolation procedures slightly and implemented a new high-throughput automated method to detect the presence of giant viruses infecting protozoa in liquid media by flow-cytometry. Flow cytometry-based methods have been previously described for their applications in studies involving pathogenic free-living amoeba (Muldrow et al., 1982; Avery et al., 1995) but have not focused on isolation-related studies. We associated enrichment methods with flow cytometry.

## Materials and Methods

### Microorganisms and Culture Conditions

For the development and validation stages, we used the *Acanthamoeba polyphaga* (*A. polyphaga*) strain Linc AP-1 as a host cell for co-culture. This strain was maintained in a 75-cm^2^ cell culture flask with 30 ml of peptone-yeast-extract-glucose medium (PYG) at 32°C, as previously described (Pagnier et al., 2013). After 48 h, the amoebae were harvested and pelleted by centrifugation. The supernatant was removed and the amoebae were re-suspended in 30 ml sterile Page’s amoeba saline (PAS). Centrifugation and suspension in PAS were repeated twice. After the last centrifugation, the amoebae were counted on counting slides (kova slides, HYCOR Biomedical, Inc., Garden Grove, CA, USA) and adjusted to 5 × 10^5^ amoeba/ml. The original strains of *A. polyphaga* Mimivirus (La Scola et al., 2003), Marseillevirus T19 (Boyer et al., 2009), and Faustovirus (Reteno et al., 2015) were used in suspension in PAS buffer. The remaining microorganisms used as cellular supports or in the gating strategy are detailed in **Table 1**. The culture conditions for the eukaryotic organisms were the same as for *A. polyphaga*, with the exception of the handling of *Vermamoeba vermiformis* (*V. vermiformis)* CD119 strain, and *Dictyostelium discoideum (D. discoideum) SG2 stain ATCC 44841*, where the amoebae were re-suspended in a newly implemented starvation medium capable of maintaining the amoeba under the best conditions for co-culture, without fast encystment as often observed in PAS. Reteno et al. (2015) used this medium for the isolation of Faustovirus. The starvation medium is composed of Yeast extract 2 gm, Glucose 18 gm, Fe (NH_4_) 2(SO_4_) 2.6 H_2_O 0.02 gm, PAS 1 L, and filtered on 0.22 μm.

**Table 1:** Microorganisms used as cellular supports or in the gating strategy.

Type	Name/strain	Growth medium T°C	Culture medium T°C	Associated Giant viruses
Amoeba	*A. polyphaga* LincAp1	PYG at 28°	PAS at 30°	Mimiviridae/Marseilleviridae
Amoeba	*A. castellanii* Neff 30010	PYG at 28°	PAS at 30°	Mimiviridae/Marseilleviridae
Amoeba	*V. vermiformis* CD119	PYG at 28°	Starvation medium at 30°	Faustovirus
Amoeba	*D. discoideum* SG2 ATCC 44841	PYG at 25°	Starvation medium at 30°	None known
Ciliate	*T. hyperangularis* X13J3	PYG at 25°	PAS at 28°	None known
Biflagellate	*P. malhamensis* ATCC 11532	PYG at 25°	PAS at 28°	None known


### Configuration of the Flow Cytometer to Detect Amoebal Lysis

To configure the flow cytometer, rinsed amoebae suspended in PAS were treated in three ways. First, at a concentration of 5 × 10^5^ amoeba/ml, amoebae were mechanically lysed by sonication. Sonication causes the amoeba to burst under the effect of ultrasound delivered by a sterile probe plugged into the falcon tube containing the amoebae. We delivered a 60-Hz frequency for 1 min. The procedure was repeated four times. These mechanically treated amoebae served as our positive control for lysis. We infected the second suspension of amoebae, at the same concentration, with Mimivirus. The third suspension of amoebae remained intact and served as a negative control for lysis. The three specimens were then dispensed to a 24-well plate at 500 μl/well. After repeatedly pipetting to detach the adherent amoebae, a 250-μl fraction of each specimen was fixed in paraformaldehyde (4%) then transferred to a suitable tube for flow cytometric analysis on the BD LSRFortessa (BD Biosciences) at 0, 24, and 48 h after infection. Data acquisition was performed using log scales for instrument scatter parameters, forward scatter (FSC) and side scatter (SSC), respectively associated with size and internal complexity of the event analyzed. Protocol threshold was adjusted on FSC parameter. Acquisition and analysis were performed using “BD FACSDiva Software.” The number of collected events was fixed for all specimens. We gated our amoeba population using the density plot representation SSC versus FSC. We performed serial dilutions from 10^6^ to 10^1^ amoebae/ml to calibrate the analyzer, to determine the reliability of the counts and to establish the limit sensitivity of detection of the flow cytometer to choose a lysis threshold that corresponds to the loss of amoebae. All amoebal counts performed by flow cytometry were also performed on kova slides to validate the counts. The quantification was also verified using counting beads (true count or cytocount “DakoCytomation,” a suspension of concentration-calibrated fluorescent microspheres). This provides the absolute count of the amoebal population using the following equation: (number of cells counted/number of Cytocount^TM^ beads counted) × Cytocount^TM^ concentration (1100 beads/μl) × dilution factor (Khan et al., 2010). We also used the Sytox nucleic acid stain for flow cytometry (Molecular Probes, Life Technologies, USA) to determine the viability of our host cells. All experiments were performed in triplicate.

### Validation of the Technique Using Samples Containing Giant Viruses

By blind culture, without microscopic observation, we tested several samples of water and soil. Some of the samples were artificially contaminated with giant virus suspensions using Mimivirus and Marseillevirus (10 μl of virus at a concentration of 10^5^ virus/ml were used to contaminate 3 ml of the sample). We contaminated 5 of 20 samples before proceeding to co-culture then for flow cytometry detection.

In a second step, we tested 80 environmental samples, including 78 samples, previously tested negative by co-culture with *A. polyphaga* and the two samples from which the Terra1 and Terra2 Mimivirus strains had previously been isolated (La Scola et al., 2010). The processing steps for isolating these Terra1 and Terra2 strains have previously been described (Pagnier et al., 2013; Yoosuf et al., 2014). For our technique, we conducted three blind co-cultures as an enrichment step; primo-culture, sub-culture, and final culture without any microscopic observation. Data acquisition on the flow cytometer was performed 24 and 48 h following the final enrichment step. The number of collected events was fixed for all samples. Uninfected amoebae were used as a negative control. They were analyzed first and used to gate the population of amoebae according to the parameters defined above. The percentage of live and dead cells was evaluated. Moreover, the presence and percentage of debris characterizing amoebal lysis was also evaluated in the FSC SSC plot. All data from LSR Fortessa were analyzed using kaluza software (Beckman Coulter). An arbitrary threshold for amoebal loss of more than 50% after 24–48 h was used to define the possible presence of a lytic agent.

### Gating of Microorganisms Using Fluorescence Staining

We used viruses of known sizes from our collection. We also used amoeba-associated chlamydiae, which can be amoebal pathogens (Greub et al., 2003). We often find chlamydiae in culture because of their resistance to the antibiotic mixtures used. Viruses and chlamydiae were analyzed on LSR fortessa. To set up Side Scatter and a 520-nm fluorescence channel on the flow cytometer, we used Megamix+ SSC beads (Biocytex, Marseille, France). This blend of size and fluorescence-calibrated beads (0.16, 0.22, 0.24, and 0.5 μm) is specially designed to evaluate and standardize Side Scatter parameters for sub-micron biological events. Virus and bacteria were gated and characterized using their original SSC/SYBR profile. Acquisition was performed using a fluorescent threshold to limit Side Scatter background noise. Sybr green was used to label DNA (SYBR Green I nucleic acid gel stain; Molecular Probes, Life Technologies, USA). The final concentration of SYBR Green I was a 10^-4^ dilution of the commercial stock solution. For an amoebal suspension at a density of 1 × 10^6^, we added 5 μl of the DiD cell labeling solution and mixed thoroughly by gentle pipetting. The cells were then incubated for 1 h at 30°C, washed three times by centrifugation and re-suspended in PAS (the purpose was to eliminate amoebal debris from the gating). We used Mimivirus, Marseillevirus, Faustovirus, and a parachlamydia-like bacterium (an intra-amoebal chlamydia isolated in our laboratory). Data for this chlamydia, that we proposed to name *Candidatus* Rubidus. massiliensis (*C.* R. massiliensis), were submitted to the EMBL database and were assigned Bio-projects number PRJEB6078; the accession numbers for the genome at EMBL are CCSC01000001–CCSC01000005) and the corresponding paper has been also submitted. A threshold on SYBR green parameter to limit background noise from side scatter was applied to detect all three potential viruses or/and bacteria in the samples. We then prepared mixes of different viral populations at various concentrations. To avoid coincidence of viral particles (i.e., two or more particles being simultaneously within the sensing zone), the samples were diluted such that the event rate was between 100 and 1000 viruses s^-1^. We tested 30 blinded mixes of viral suspensions in order to validate the proof of concept. After optimizing the gating strategy, we screened all of the previously tested real environmental samples that showed host cell lysis and contained mixture of viruses. Ten μl of the culture supernatant after host lysis detection on the sample was diluted to 1/10^4^ in PAS, then stained with SYBR green at 30°C over night. We then performed gating to distinguish between a single virus, a viral mixture and bacteria. The results of the gating were confirmed by targeted PCR of the different giant viruses (Ngounga et al., 2013).

### Automation and Adaptation of the Method to Other Protozoa

We adapted our method to all protozoa enumerated in **Table 1**. All tests performed during the developmental stage with *A. polyphaga*, were carried out on each protozoon. We also tested protozoan survival in the antibiotic and antifungal mixture before the gating stage and the use of the protozoa as cell hosts in culture. Initially, all experiments were performed in 24-well plates. Because our purpose was high-throughput detection, we then adapted our technique to 96-well plates. We optimized the concentration and volume of amoebae in these plates (150 μl of amoeba suspension at a concentration of 5 × 10^5^ amoeba/ml for *Acanthamoeba castellanii* (*A. castellanii)*, *A. polyphaga*, *Poterioochromonas malhamensis* (*P. malhamensis)*, *Tetrahymena hyperangularis* (*T. hyperangularis)*, and 150 μl of amoebal suspension at a concentration of 10^6^ amoeba/ml for *V. vermiformis*, and *D. discoideum*), and used 100 μl of each sample. Following optimization in 96-well micro-plates, we integrated a high-throughput sampler (HTS) from BD Biosciences with the LSRFortessa analyzer. The HTS loads samples from the 96-well plates and performs fully automated acquisition within 40 min. Under high pressure and after several mixes, the HTS is easily capable of re-suspending the adherent amoebae in the plates. The above experiments were performed in triplicate on these 96-well plates to confirm the reliability of the technique when optimized for micro-plates. The same principle of automation was applied to the gating strategy. We adapted the same experiment principle performed in tubes to 96-well micro-plates.

### Automated Sample Screening

For sample testing, we used our high-throughput method to launch high-speed screening for giant viruses in environmental and clinical samples. There were 735 bronchoalveolar lavages (BALs) from the intensive care unit of the La Timone Marseille healthcare center, France. We collected 250 samples of sewage (80 from Marseille-France, 50 from Dakar-Senegal, 70 from Tripoli Lebanon, and 50 from Guyana DOM France), 1025 seawater and soil samples (400 seawater and 50 soil samples from Marseille France, 400 seawater samples from La Ciotat, Bandol, France, 160 seawater and 15 soil samples from Brittany France). All samples were tested on four protozoa (*A. polyphaga*, *V. vermiformis*, *T. hyperangularis*, and *P. malhamensis*). The enrichment step used antibiotic and antifungal mixtures containing 10 μg/ml vancomycin (Mylan, Saint-Priest, France), 10 μg/ml imipenem, 20 μg/ml ciprofloxacin (Panpharma, Z.I. du Clairay, France), and either a fungicide [30 μg/ml thiabendazole (TBZ)] or an antifungal (30 μg/ml Fungizone, Bristol Myers Squibb, Rueil-Malmaison, France), as previously described (Pagnier et al., 2013). After host cell lysis detection, and preliminary gating characterization, lytic agents were then identified using MALDI-TOF MS for bacteria (Seng et al., 2010, 2013) and a specific primer-probe system for giant viruses as previously described (Pagnier et al., 2013).

### Ethics Statement

LBA samples were received from an anonymized patient file for the research of emerging pathogens (IFR48- No 13-031).

### Statistics

We used the R software package (R Core Team, 2013), a language and environment for statistical computing and analysis version 3.0. Spearman correlations were used to look for a link between kova slides counts and flow cytometry counts. A *p*-value of <0.05 was considered statistically significant. Experiments were performed in triplicate.

## Results

### Detection of Amoebal Lysis by Flow Cytometry

For flow cytometry configuration, we observed a significant decrease (90%) in the percentage of amoebae and an increase in the number of debris following mechanical lysis and infection with Mimivirus at 24 and 48 h compared to the percentage of control amoebae. The counts on kova slides and by flow cytometry are correlated (Spearman *r* = 0.97, *p*-value < 0.001). The detection limit of the FACS was of 10^2^ amoebae/ml, which is sufficient for detecting the arbitrary threshold of an amoebal loss greater than 50% after 24 h.

### Technique Concept Validation

**FIGURE 1:**
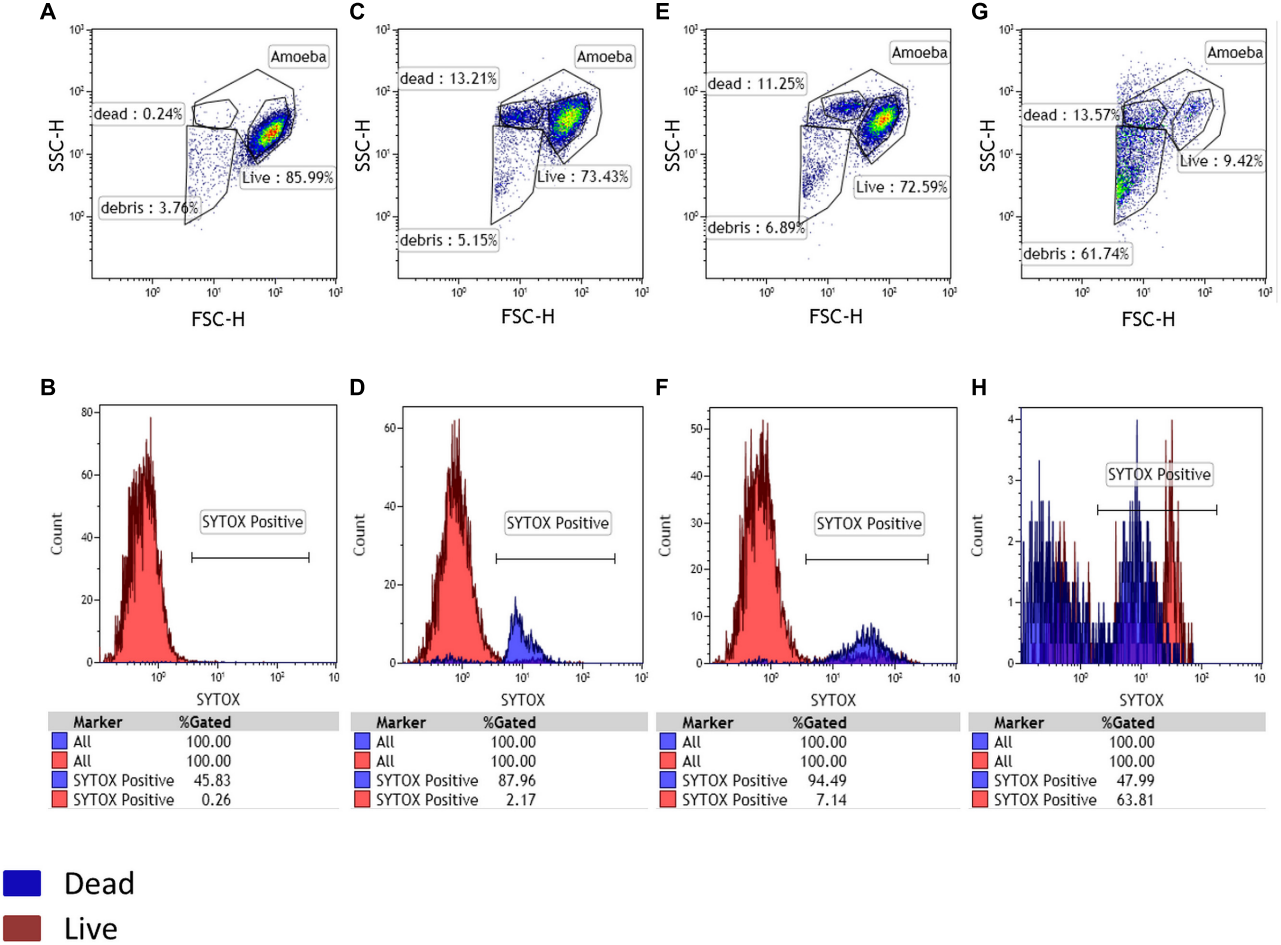
**Detection of potential amoebal infection.** A living amoebal population was used as a negative control with Sytox staining. Three gates were designed in the FSC-SSC plot, potentially corresponding to live cells, dead cells and debris. At 0 h the negative control and sample showed the same gated populations **(A,B)**. After 24 h of culture, a physiologic increase in dead cells was observed for both the negative control and test samples **(C,D)**. The appearance of a large population of debris confirmed a potential infection after 48 h of culture and a larger part of dead cells in the tested samples than in the negative control showing no substantial changes in the gated populations (**E,F** representing the negative control panel after 48 h and, **G,H** representing the sample containing Mimivirus after 48 h).

During the artificial contamination tests, we managed to detect all five artificially contaminated samples among the 20 tested samples. At 0 h, the negative control used as a reference population contained 86% of amoebae. The same percentage was equally observed for the other samples at this time point. It is important to note the physiologic increase in sytox positive dead cells for both control and tested samples at 24 h post-culture. At 48 h we still had our negative control with no substantial changes. By contrast, the positive control showed an almost complete loss of gated amoeba with the highest percentage of debris. These results were obtained by applying size and granularity parameters (FSC and SSC channels). The sytox nucleic acid stain was used to confirm and validate our gatings (**Figure 1**). For all real environmental samples the results obtained by our new high-speed method were identical to those obtained previously by the traditional co-culture method (Pagnier et al., 2013). We detected the two positive samples among the 80 tested samples. PCR for giant viruses, targeting pol B DNA, was performed to identify the sequences corresponding to Terra1 and Terra2 viruses (La Scola et al., 2010). In addition, a single sample, previously identified as negative in co-culture, appeared positive by cytometry. For that sample, additional investigations, such as staining (Gram, Giminez, Hemacolor, negative staining) and a PCR system targeting giant viruses (Ngounga et al., 2013) were performed on this sample, and demonstrated a newly isolated Mimivirus of genotype C (Colson et al., 2012), submitted under bioproject PRJNA278018.

### Results of the Gating Strategy

**FIGURE 2:**
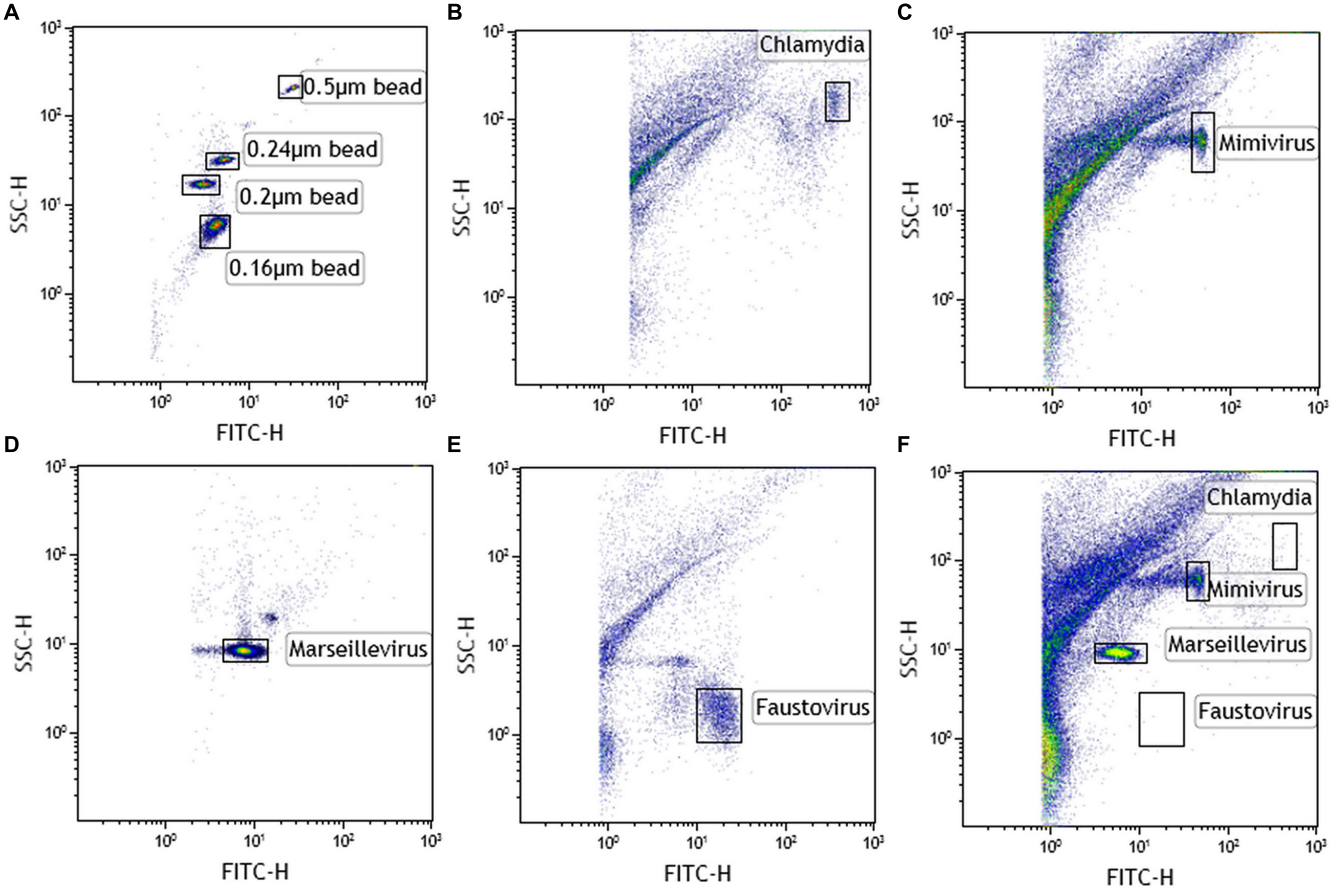
**Cytometric analysis of amoebal pathogens: giant viruses and chlamydiae.** **(A)** Using Megamix+ SSC beads (0.16-, 0.2-, 0.24-, and 0.5-μm fluorescent beads), LSR fortessa was easily set up for SSC, and a 520 nm Fluorescent channel (for SYBR DNA virus labeling) was used for virus range analysis. Fluorescence threshold was set to limit background noise of the instrument. Each microorganism was first analyzed alone to clearly define virus gates [**(B)** chlamydia, **(C)** Mimivirus, **(D)** Marseillevirus, and **(E)** Faustovirus]. Thanks to the wide resolution of LSR fortessa in SSC and the clear DNA content between the different biological events, four different gates were created. Due to the high concentration of virus in the supernatant after amoebal burst, a virus was considered present if its concentration was more than 10^3^ virus/μl (counting beads). **(F)** Viral mixture of Mimivirus and Marseillevirus, detected in the same sample.

Single virus/bacterium analysis allows SSC/DNA content profile to be determined. After setting up the instrument using Megamix and SSC beads (**Figure 2A**), we found that *Chlamydia* gave an SSC signal close to the 0.55 μm Megamix and a high SYBR labeling, as expected (**Figure 2B**). Mimivirus with SSC beads showed intermediate SYBR labeling between 0.24 and 0.55 μm (**Figure 2C**). Marseillevirus with SSC showed the lowest SYBR signal of the four species at 0.16 μm bead SSC signal (**Figure 2D**). Finally, Faustovirus showed minimal SSC (below the 0.16 μm bead) but a higher DNA content than Marseillevirus (**Figure 2E**). A linear correlation was found between green fluorescence intensity and genome size for giant viruses (*r*^2^ = 0.98). We confirmed our findings of the presence of a viral mixture of Mimivirus and Marseillevirus in natural environmental samples by this gating strategy (**Figure 2F**). All of the samples that were previously tested in culture and that showed host cell lysis were screened by the gating technique. We were able to detect all of the viral or bacterial populations in the samples that were amplified by the enrichment step. All of the results were confirmed by a giant virus-specific PCR system (Ngounga et al., 2013).

### Results of the Method Automation and Sample Screening

After adapting our technique to all of the protozoa types used as host cells, the automation allowed us to test 96 samples in less than 40 min. Of the 735 BAL samples tested on *A. polyphaga*, *V. vermiformis*, *T. hyperangularis*, and *P. malhamensis*, three led to *A. polyphaga* lysis detection. We identified three bacteria (*Pseudomonas aeruginosa, Acinetobacter baumannii*, and *Stenotrophomonas maltophilia*), but no giant viruses. Among the sewage samples carried out on *A. polyphaga*, 10 led to detection of amoebal lysis. The gating strategy revealed the presence of viral mixtures in many samples. PCR performed on positive wells confirmed the gating strategy results and revealed the simultaneous presence of a Mimivirus of genotype A, a Mimivirus of genotype C and a Marseillevirus in one sample from Marseille. A second sample contained a Mimivirus of genotype A from Lebanon, and the eight remaining samples contained a mix of Mimivirus genotypes A and C, 3 from Dakar and 5 from Marseille. For the same sewage samples inoculated on *V. vermiformis*, five were positive and we isolated a new giant virus specific for *Vermamoeba*, named Faustovirus E12 (Reteno et al., 2015). Seven other viruses belonging to the same family were also isolated from the different sewage samples (3 from Marseille, 3 from Dakar, and 1 from Lebanon; the genomic data corresponding to these isolates were submitted to the EMBL database and were assigned Bio-projects numbers respectively: E24: PRJNA279158, E23: PRJNA279157, E9: PRJNA279166, D3: PRJNA279161, D5: PRJNA279159, D6: PRJNA279164, Liban: PRJNA279165). No lysis was detected on the other tested protozoa. The results of this screening are summarized in **Table 2**.

**Table 2:** Isolates from environmental and clinical samples represented in collection area and with the different used host cells.

Sample type	Country	*A. polyphaga*	*V. vermiformis*	*T. hyperangularis*
				*P. malhamensis*
Sewage	France	Bacteria: 0	Bacteria: 0	Bacteria: 0
	*N* = 80 Marseille	Virus: 6 from Marseille	Virus: 4 from Marseille	Virus: 0
	*N* = 50 Guyana	E5^∗∗∗^	Faustovirus E12	
		E8, E20, E19, E31, E25^∗∗^	Faustovirus E9	
			Faustovirus E23	
			Faustovirus E24	
	Dakkar	Bacteria: 0	Bacteria: 0	Bacteria: 0
	*N* = 50	Virus: 3	Virus: 3	Virus:0
		D5, D8, D15^∗∗^	Faustovirus D3
			Faustovirus D5	
			Faustovirus D6	
	Lebanon	Bacteria:0	Bacteria: 0	Bacteria: 0
*N* = 70	Virus: 1	Virus: 1	Virus: 0
		Liban 10: Mimivirus A	Faustovirus Liban 4	
Soil	France	Bacteria: 0	Bacteria: 0	Bacteria: 0
	Marseille	Virus: 0	Virus: 0	Virus: 0
	*N* = 50			
	Brittany-France	Bacteria: 0	Bacteria: 0	Bacteria: 0
	*N* = 15	Virus: 0	Virus: 0	Virus: 0
Sea water	France	Bacteria: 0	Bacteria: 0	Bacteria: 0
	Marseille, La ciotat	Virus: 0	Virus: 0	Virus: 0
	*N* = 800			
	Brittany-France	Bacteria: 0	Bacteria: 0	Bacteria: 0
	*N* = 160	Virus: 0	Virus: 0	Virus: 0
BAL	France	Bacteria: 3	Bacteria: 0	Bacteria: 0
	Timone	*Pseudomonas aeruginosa,*		
	Health care Centre	*Acinetobacter baumannii,*		
	*N* = 735	*Stenotrophomonas maltophilia,*		
		Virus: 0	Virus: 0	Virus: 0


*N = number of collected samples. ^∗∗^Mix Mimivirus A, Mimivirus C. ^∗∗∗^Mix Mimivirus A Mimivirus C and Marseillevirus.*

## Discussion

The study of giant viruses is a newly emerging field in virology. Metagenomics data have suggested the ubiquity of giant viruses not only in the environment (Claverie, 2004; Monier et al., 2008a,b; Claverie et al., 2009) but also in humans (Colson et al., 2013). We must therefore search for these viruses at large scales. This high-throughput sample screening was almost impossible to conduct with our usual techniques of co-culture used by almost all the scientific committee nowadays, especially for non-adherent or motile protozoa. The method we developed in this paper provides the necessary characteristics for high-throughput analyses by allowing host cell lysis detection in micro-assays, followed by a gating strategy capable of preliminary characterization of the infectious agent responsible for host cell lysis. The method was developed in three stages. The first step included the calibration and configuration procedures performed with (*A. polyphaga*) to detect amoebal lysis. This was followed by the validation stage, then by the automation that simplified the enrichment step. We then adapted this technique to other types of protozoa, which we think that may harvest other giant viruses, to finally test hundreds of environmental and clinical samples for the presence of viruses.

Detecting the loss of the amoeba-gated population represents a signal for the presence of a pathogenic agent responsible for the cytopathogenic effect and amoebal lysis. It is thus important to accurately determine the percentage of each population using the best possible gating. These percentages can vary, so it is important to always have a negative control containing the host cell under the most favorable culture conditions, which will be used as the reference population for the rest of the samples. The Sytox nucleic acid stain can always help us evaluate the viability of our amoebae.

Following the successful results from the developmental stage, the proof of concept was validated by detecting not only known viruses from both artificially contaminated samples and environmental samples but also a new virus, previously undetected by the traditional methods of co-culture with *Acanthamoeba* (Pagnier et al., 2013). The isolation of this later strain suggests a higher sensitivity of our new technique. This method allowed the rapid isolation of more than 10 new strains of giant viruses. No giant virus was found in the BAL samples, clearly confirming the rare direct evidence for detecting giant viruses in BAL, at least using *A. polyphaga* as a support for the culture. We did, however, detect three bacterial strains. These bacteria were isolated from intensive care unit patients because they were resistant to the antibiotic mixtures used during the different stages of the co-culture. The discovery of a new host-specific giant virus (Reteno et al., 2015) is good proof that other protozoa species should be tested as hosts cells capable of harvesting giant viruses. We also succeeded in isolating 7 new isolates of this newly discovered Faustovirus.

Cytometric analysis has several advantages over current isolation methods. Using this system, it is possible to rapidly quantify amoebae and identify the presence of pathogenic microorganisms, such as giant viruses, in any type of sample.

A major benefit of this high-throughput method is its automation. This automated method largely reduces the time required to screen 1000s of samples for giant viruses, especially as data acquisition on the flow cytometer does not exceed half an hour. The cytometer is able to detect lysis at an early stage, which is better than microscopic observation. Other advantages of flow cytometry over routine microscopic observation include a high degree of statistical precision due to the large numbers measured, the high-speed gating and identification of the amoebal status, along with the elimination of subjectivity.

The production of amoebae in culture was one of the limiting factors of the old techniques (Boughalmi et al., 2012; Pagnier et al., 2013) especially as they remain time consuming and not amenable to automation. In this respect, the enrichment steps using micro plates greatly reduced culture preparation time. For example, a 96-well plate of samples requires four times fewer amoebae than the routine culture performed in 12- or 24-well plates. All of these advantages made our method a compatible tool for high-throughput platforms. Although the *Acanthamoeba* species allowed the isolation of giant viruses, we know that other protozoa are capable of growing similar viruses, where *Cafeteria roenbergensis*, a marine biflagellate was isolated with its associated virus CroV (Garza and Suttle, 1995; Fischer et al., 2010). It is the same case for PgV-16T (*Phaeocystis globosa* virus) that infects a nanoplanktonic marine alga (Santini et al., 2013). The isolation of the latter two viruses was the consequence of their associated protozoan isolation and was not from direct research using co-culture. Because the protozoan world is vast, the diversity of giant viruses is most likely vast as well, and with the previous culture techniques, our research and knowledge was limited. Indeed, several electron microscopy photos suggest that some relatively large viruses showing similar morphology to the NCLDV family are present within numerous other eukaryotic microorganisms, including the parasites *Giardia* and *Blastocystis* (Stenzel and Boreham, 1997).

The method we designed is readily amenable to other protozoa, especially those capable of growing only in liquid media, such as ciliates and flagellates. Thus, it is adapted to other types of protozoa and allows us to enhance the number of host cells for the screening of diverse environmental or human samples. The limitation of the method comes from the detection limit of the cytometer where we cannot go below 10^2^ cells/ml. The method still requires a culture enrichment step that needs the host species to be cultivated in sufficient number, which is a limiting factor with species such as *Giardia* and *Blastocystis*. We are aware that it will be hard to cover all types of protozoa and we will try to improve the detection sensibility in order to be able to detect lysis on fastidious and slowly growing host cells.

The gating strategy will detect the nature of the lytic agent (virus, bacteria, or mixture in the same sample) without losing time, especially compared to standard staining techniques such as Gram, hemacolor, dapi, etc. We developed and applied the gating strategy using SYBR Green 1 inspired from previous works (Marie et al., 1999; Brussaard et al., 2000; Brussaard, 2004). Based on the different fluorescence profiles that we have already characterized for each gated microorganism, we can easily discriminate between all known giant viruses that we could isolate after detection of lysis. The gating strategy will limit us to only gated microorganisms, so it would be interesting to enlarge the gating data by discovering more giant viruses. Many samples contained mixtures of viruses. For this, we believe that the adaptation of the method to a real FACS sorting technique will be the best strategy for better preliminary characterization of new isolates and especially for separating viral mixtures in order to allow a better comprehensive pangenomic study of these virus families and their ecosystems. The integration of cell sorting to purify cell populations enables more facilities to isolate, produce and clone giant viruses, while offering the possibility of studying the infectious stage before amoeba burst to possibly identify non-lytic viruses or endosymbionts and this by focusing on the cell host nuclear changes and viability biomarkers.

## Conflict of Interest Statement

The authors declare that the research was conducted in the absence of any commercial or financial relationships that could be construed as a potential conflict of interest.
